# Evaluation of the anti–depressant activity of *Myristica fragrans* (Nutmeg) in male rats

**Published:** 2012

**Authors:** Ghulam Moinuddin, Kshama Devi, Deepak Kumar Khajuria

**Affiliations:** 1 *Department of Pharmacology, Al-Ameen College of Pharmacy, Near Lalbagh Main Gate, Bangalore-560027. India*

**Keywords:** Antidepressant activity, Imipramine, *Myristica fragrans*

## Abstract

**Objective: **The present study was undertaken to evaluate anti-depressant activity of *Myristica fragrans* (MS).

**Materials and Methods: **Male Wistar rats were subjected to imipramine and herbal extract of MS for their antidepressant activity using Forced Swimming Test (FST), Reserpine Reversal Test (RRT), Haloperidol-Induced Catalepsy (HIC), and Pentobarbitone Sleeping Time (PST).

**Results:** Administration of MS and imipramine revealed a statistically significant reduction in immobility time in FST, RRT, and protection against HIC, compared to the control group. However, there was no significant potentiation of PST.

**Conclusion: **Our study demonstrated the potential antidepressant activity of MS.

## Introduction

Depression is considered as an affective disorder with a prevalence of approximately 5% in the general population. It is characterized by change in mood, lack of interest in the surroundings, psychomotor retardation, and melancholia. It has been estimated that 5.8% of men and 9.5% of women experience a depressive episode in their lifetime and suicide is one of the most common outcomes of depression (WHO, 1998 ▶; Stahl, 1996 ▶; Richelson, 2001 ▶). Depression is a common, debilitating, life threatening illness with an increasing morbidity and mortality. Furthermore, the World Health Organization (WHO) reported that depression is the fourth leading causes of disability worldwide (WHO, 2001 ▶). 

Despite the developments in pharmacotherapy of depression, this disorder often goes undiagnosed and untreated in many patients. Although the drugs provide some improvement in the clinical condition of patient, it is at a cost of having to bear the burden of their adverse effects (Stahl, 1996 ▶; Tripathi, 2008 ▶; Hardman et al., 2007 ▶). This is further complicated by the difficulty in predicting the patients’ response to treatment. It has been reported that only two out of three patients respond to any given antidepressant treatment, and of these, one would probably have responded to placebo alone (Stahl, 1996 ▶; Walker and Edward 1999 ▶). The exact etiology of depression still remains obscure, but the most popular theory is the decrease in the neurotransmitter levels in the brain. However, recent studies have also shown the involvement of oxidative stress in the phenomenon (Sarandol et al., 2007 ▶; Ibrahim et al., 2007 ▶).

Many plants have been used in the traditional medicine for the treatment of depression and associated disorders (Sembulingam et al., 1997 ▶). Because of the lacunae in the current treatment options, there has been an increase in the number of patients turning toward alternative and complimentary systems of medicine to obtain symptomatic relief. 

Nutmeg (*Myristica fragrans*; MS) is widely used in a variety of ways and for various purposes. Dating back to the 16^th^ century, nutmeg has been known for its psychoactive properties, which include anxiogenic and hallucination (Brunner et al., 1993 ▶; Forrester, 2005 ▶). Medicinally, nutmeg is known for its anti-inflammatory and anti-thrombotic (Olazide et al., 1999), as well as anti-rheumatic, carminative and stimulant properties (Prabuseenivasan et al., 2006 ▶). In pregnancy and lactation, nutmeg is used in traditional medicine practice for antenatal and postnatal treatment (Lavy, 1987 ▶). 

These data confirm that MS modulate the physiology of the CNS. The purpose of this experiment was to test the antidepressant effect of MS on rats using Forced Swimming Test (FST), Reserpine Reversal Test (RRT), Haloperidol-Induced Catalepsy (HIC), and Pentobarbitone Sleeping Time (PST).

## Materials and Methods

**Animals**

Healthy adult male Wistar rats were used. Animals were kept under standard laboratory conditions. Commercial pellet diet (Amruth Limited, India) and water were provided *ad libitum*. The experimental protocol was approved by the Institutional Animal Ethics Committee and by the animal regulatory body of the government (Al-Ameen College of Pharmacy, India. Reg. No. 83/1999/CPCSEA).

**Plant materials**/**chemicals**

MS extract was obtained from Sami Chemicals and Extracts Pvt. Ltd, Bangalore, India. The standard drug imipramine was procured from Torrent Pharmaceuticals, India. Plant extract was suspended in 2% Tween 80 immediately prior to the administration. Drugs used in different animal models of depression were Haloperidol (Searle India Ltd., India), Reserpine (Loba-Chemie Industrial Co. India), and Pentobarbitone sodium (John Baker mc, Colorado USA). Weighed quantity of MS extract was suspended in distilled water using 2% Tween 80 and administered orally, in a volume of 1 ml/ 100 g, one hour before the experiment. Imipramine (15mg/kg) was administered *i.p.* one hour before experimentation. Reserpine (2 mg/kg) was suspended in distilled water using 2% Tween 80 and administered *i.p.* 24 hours before the experiment. Haloperidol (1mg/kg) was dissolved in distilled water and injected *i.p.* 30 min before experimentation in a volume of 0.5 ml/100 g. Sodium pentobarbitone (30 mg/kg) was dissolved in warm distilled water and administered *i.p.* in a volume of 0.5 ml/100 g. 

**Experimental protocol**

Animals were randomly divided into three groups consisting of six rats each. Control group, received calculated dose of vehicle (l ml/100g) oral route. Standard group received imipramine 15 mg/kg/*i.p.* (Kumar et al., 1999). The MS group received MS 500 mg/kg/*p.o. *The dose of MS was calculated based on the dose response study carried out in our previous work.

**Forced swimming test (FST)**

In FST, measurement of immobility time was carried out by observing the motor activity of the rat, which was placed in a pool of water. A glass cylinder, 25 cm in diameter, height 40 cm, was filled with water to a height of 34 cm. The temperature of water was 23±1º C. After 15 minutes (pretest session), the rats were towel dried and kept for 15 minutes in a heated enclosure (32° C). Twenty-four hours later, the animals were exposed again to the conditions outlined above and the total immobility time during 5 minutes period was recorded (test session). Drug/vehicle was administered to the animals 30 minutes before the FST. The time spent immobile within 5 minutes was recorded. Immobility time is the time during which the animal floated on the surface with front paws together and made only those movements which were necessary to keep afloat. Shorter immobility time is an indicator of the stronger antidepressant effect of the tested substance (Porosolt, 1978 ▶).

**Reserpine reversal test (RRT)**

In RRT, the animals of all groups received calculated dose of reserpine suspended in distilled water in a dose of 2 mg/kg intraperitonially 24 hours before experimentation. The different experimental groups received their respective treatments and the reduction in reserpine induced immobility period was measured in individual groups one hour after the respective treatment. The time spent immobile within five minutes was recorded. Reserpine was administered to the rats (2mg/kg/*i.p.*), after 24 hours, the extract was administered to the animals by oral route and reduction in reserpine induced immobility period was measured one hour after drug administration. The time spent immobile within five minutes was recorded (Costa et al., 1960 ▶).

**Haloperidol-induced catalepsy (HIC)**

In HIC, the different experimental groups received the respective treatments; calculated dose of haloperidol was administered *i.p.* one hour after the administration of their respective treatments. 

After administration of haloperidol, 1 mg/kg, severity of catalepsy was measured every 30 minutes up to a total duration of 3 hours. Catalepsy of an individual rat was measured in a stepwise manner by an scoring method. The method assessed the ability of an animal to respond to an externally imposed posture in the following manner: Step I: the rat was taken out of the home cage and placed on a table. If the rat failed to move when touched gently on the back or pushed, a score of 0.5 was assigned. Step II: The front paws of the rat were placed alternately on a 3 cm high block. If the rat failed to correct the posture within 10 seconds, a score of 0.5 for each paw was added to the score of the step I. Step III: the front paws of the rat placed alternately on a 9 cm high block. If the rat failed to correct the posture within 15 seconds, a score of 1 for each paw was added to the scores of step I and II. Thus, for an animal, the highest score was 3.5 (cut off score) and reflects total catalepsy (Khisti et al., 1997).

**Pentobarbitone sleeping time (PST)**

In PST test, different experimental groups received the respective treatments. One hour later, calculated dose of sodium pentobarbitone (30 mg/kg/*i.p.*) was administered to the animals in all groups. The time of onset of action was noted as the animal loses its righting reflex, that it falls asleep. The animals were placed on their backs leaving sufficient space in between two animals. The time of recovery from sleep as the animal turns to recover its normal posture was recorded (Das and Guha, 2007 ▶).

**Statistical analysis**

Data are expressed as mean±SEM. The results were subjected to one-way analysis of variance (ANOVA), followed by Dunnet’s multiple comparison test to compare the treatment groups with control group.

## Results

**Forced swimming test**

After administration of a single oral dose, statistically significant decrease (p<0.001) in the immobility time in FST was observed with MS 500 mg/kg, when compared to the control group. The extent of decrease in immobility time in case of MS was found to be very higher than that of imipramine (Table 1).

**Table 1:** Effect of acute administration of *Myristica fragrans* and imipramine on forced swimming induced immobility in rats.

**Treatment**	**Dose (mg/kg)**	**Immobility(Sec)**
Vehicle	-	223.6±3.4
Imipramine	15	161.6±6.1***
*Myristica fragrans*	500	163.0±11.7***

Values are expressed as mean±SEM, n=6,

*** p<0.001comparet to vehicle.One Way Analysis of Variance (ANOVA) followed by Dunnet’s t test.

**Reserpine reversal test**

A statistically significant decrease (p<0.001) of immobility time in Reserpine Induced Immobility was observed after the administration of a single oral dose of MS, as compared with control group. The extent of decrease in immobility time in case of MS was found to be very higher than that of imipramine (Table 2).

**Table 2:** Effect of acute administration of *Myristica fragrans* and imipramine on Reserpine Induced Immobility in Rats

**Treatment**	**Dose (mg/kg)**	**Immobility(Sec)**
Vehicle	-	280.8 ± 6.8
Imipramine	15	122.1±13.1***
*Myristica fragrans*	500	147.5±15.7***

Values are expressed as mean±SEM, n=6,

*** p<0.001 comparet to vehicle..One Way Analysis of Variance (ANOVA) followed by Dunnet’s t test.

**Haloperidol induced catalepsy**

MS provides highly significant (p<0.001) protection against haloperidol (1 mg/kg) induced catalepsy as compared to control, up to 180 minutes after intraperitoneal injection of haloperidol. 

**Table 3:** Effect of acute administration of *Myristica fragrans* and imipramine on Halaoperidol Induced Catalepsy in Rats

**Treatment**	**Dose (mg/kg)**	**Score of Catalepsy**					
		**Time (Min.)**					
		**30**	**60**	**90**	**120**	**150**	**180**
Vehicle	-	0.5±0.1	2.7±0.2	3.2±0.2	3.4±0.1	3.5±0.0	3.3±0.2
Imipramine	15	0.1±0.1*	0.7±0.3	1.0±0.3	1.5±0.3	1.6±0.3	1.6±1.4**
*Myristica fragrans*	500	0.2±0.1	0.5±0.2	0.2±0.2	1.0±0.2	1.2±0.2	1.2±0.2***

Values are expressed as mean±SEM, n=6,

* p<0.05,

** p<0.01,

*** p<0.001 comparet to vehicle.One Way Analysis of Variance (ANOVA) followed by Dunnet’s t test.

However, MS showed statistically significant inhibition of haloperidol induced catalepsy at 30 minutes after injection of haloperidol. The protection given by MS was more than that of imipramine (Table 3). 

**Pentobarbitone sleeping time**

Compared to the control group, no significant potentiation of pentobarbitone-induced loss of righting reflex was observed after pretreatment with MS extract (p<0.05). However, treatment with imipramine significantly increased (p<0.001) the pentobarbitone induced loss of righting reflex (Table 4).

**Table 4:** Effect of acute administration of *Myristica fragrans* and imipramine on Pentobarbitone Induced Sleeping Time in Rats

**Treatment**	**Dose (mg/kg)**	**Sleeping Time (Min)**
Vehicle	-	95.3±7.1
Imipramine	15	137.1±11.2***
*Myristica fragrans*	500	101.1±1.7

Values are expressed as mean±SEM, n=6,

*** p<,0.001 comparet to vehicle.One Way Analysis of Variance (ANOVA) followed by Dunnet’s t test.

## Discussion

Most of the drugs that are currently being used in the treatment of depression have adverse effects that affect the quality of life of the patients. This leads to patients’ non-compliance to medications, which further complicates the problem (Stahl, 1996 ▶; Tripathi, 2008 ▶; Hardman et al., 2007 ▶). In Ayurveda, number of single and multi drug formulations from plant origin are used in the treatment of psychiatric disorders (Tipathi, 2008 ▶; Sembuligam et al., 1997 ▶) and are claimed to have lesser side effects than conventional allopathic drugs. 

Development of immobility when rodents are placed in an inescapable cylinder of water during FST reflects the cessation of their persistent escape-directed behavior. Conventional antidepressant drugs reliably decrease the duration of immobility in animals during these tests. This decrease in duration of immobility was considered to have a good predictive value in the evaluation of potential antidepressant agents (Porosolt, 1981 ▶). 

In the present study, MS caused a significant decrease in immobility induced by FST and RRT. The decrease in immobility was comparable to that produced by the standard antidepressant drug, imipramine. Neuroleptic-induced catalepsy has been linked to blockade of postsynaptic striatal dopamine D1 and D2 receptors (Samberg, 1980 ▶). In addition, many preclinical and clinical studies have also proposed the reactive oxygen species as cause of haloperidol-induced catalepsy (Polydoro et al., 2004). In the present study, MS showed statistically significant inhibition of haloperidol induced catalepsy. Thus, the anti-cataleptic effect of MS might be due to both its dopamine facilitatory and anti-oxidant properties. The anti-cataleptic activity of MS was comparable to that of standard antidepressant drug, imipramine. 

However, MS did not show any significant potentiation of the loss of righting reflex as induced by pentobarbitone. The barbiturates are known to induce sleep in man and animals by depressing the central nervous system by high affinity binding to specific macromolecules within CNS. These barbiturates binding sites are closely associated with the GABAergic receptors. GABA-chloride ionophores appear to prolong rather than intensify GABA effects and it can be suggested that the antidepressant effect of the MS extract observed in the present investigation could not be possibly mediated by GABAergic receptors. 

Since MS exhibited better/equivalent effect to that of imipramine at the dose tested in FST, RRT, and HIC in rats, the drug is rated to possess potentially significant antidepressant activity.

The most prevalent theory for the pathogenesis of depression is “Monoamine hypothesis”. Functional deficiency of central monoamines such as noradrenaline, 5-hydroxytryptamine, and dopamine are responsible for the symptoms of depression (Schildkrant, 1965 ▶). Many currently used antidepressants act by increasing the concentration of these neurotransmitters in the brain (X-Malbey and Rosenweig-Lipson, 2005 ▶; Richelson, 2002 ▶). Therefore, the antidepressant-like activity of MS might be due its modulatory effect on central monoamines. Data obtained in our preclinical study allows us to propose this plant as an excellent candidate for isolating new substances with potential antidepressant activity. 
